# Socio-economic inequalities in smoking prevalence and involuntary exposure to tobacco smoke in Argentina: Analysis of three cross-sectional nationally representative surveys in 2005, 2009 and 2013

**DOI:** 10.1371/journal.pone.0217845

**Published:** 2019-06-07

**Authors:** Marilina Santero, Santiago Melendi, Akram Hernández-Vásquez, Vilma Irazola

**Affiliations:** 1 Departmento de Enfermedades Crónicas, Instituto de Efectividad Clínica y Sanitaria (IECS), Buenos Aires, Argentina; 2 Universidad San Ignacio de Loyola, Vicerrectorado de Investigación, Centro de Excelencia en Investigaciones Económicas y Sociales en Salud, Lima, Peru; Ben-Gurion University of the Negev Faculty of Health Sciences, ISRAEL

## Abstract

**Background:**

Understanding patterns of socio-economic inequalities in tobacco consumption is key to design targeted public health policies for tobacco control. This study examines socio-economic inequalities in smoking and involuntary exposure to tobacco smoke between 2005 and 2013.

**Methods:**

Data were derived from the Argentine National Risk Factors Surveys, conducted in 2005, 2009, and 2013. Two inequality measures were calculated: the age-adjusted prevalence ratio (PR) and the disparity index (DI). Educational level, household income per consumer unit and employment status were used as proxies for socio-economic status (SES). Generalized linear models were used in the analysis.

**Results:**

Prevalence of smoking decreased from 29.7% to 25.1% between 2005 and 2013, mainly in women (p<0.001). Despite the overall prevalence reduction, socio-economic inequalities in smoking persisted. For both men and women, the DI was moderately high for smoking (14.47%-33.06%) across the three surveys. In men, the PR indicated a higher smoking prevalence for lower educational levels and lower household income throughout the analyzed period. In women, unlike previous years, the 2013 survey showed disparity related to unemployment. Involuntary exposure to tobacco smoke in 2013 was associated with educational level and household income, with lower involuntary exposure among those with higher SES.

**Conclusions:**

While overall smoking rates have decreased in Argentina, socio-economic disparities related to tobacco smoking persist. Comprehensive tobacco control programs targeted to address these inequalities are essential in developing strategies to reduce health disparities in tobacco-related diseases.

## Introduction

While great progress has been made towards tobacco control throughout the world, smoking persists as one of the major causes of premature mortality and morbidity and, therefore, constitutes a global public health priority. Smoking remains among the leading five risk factors of disability-adjusted life years (DALYs) for 109 countries [1]. The World Health Organization (WHO) estimates that about 7 million people worldwide die each year from smoking-related diseases, a twofold increase since 2000 [2]. More than 6 million of those deaths are the result of direct tobacco use while around 890,000 are the result of involuntary exposure to tobacco smoke [3]. Additionally, it is projected that by 2030, 80% of all tobacco deaths will occur in developing countries [4].

In Argentina, several studies indicate that tobacco is the second most consumed psychoactive substance, behind alcohol [5]. According to the "Report on Drug Use in the Americas, 2015”, carried out by the Organization of American States (OAS), Argentina constitutes the second country to present the highest tobacco consumption in the region, after Chile. Multiple surveys, with diverse methodologies, agree that smoking prevalence among males is higher than among females, and that young people aged 25 to 34 years show the highest level of consumption [6–8]. Tobacco causes more than 100 deaths per day (40,000 per year, 6000 due to secondhand smoke). Every year, 998,881 years of life are lost from premature death and disability. The estimated cost of treating tobacco-attributable health problems is 270 billion United States Dollars (USD) and tobacco taxes only cover 67.3% of this expense [9].

The WHO Framework Convention on Tobacco Control (FCTC) entered into force in 2005 and has been ratified by 180 parties, formalizing the global commitment to reducing smoking. [10,11]. To address the harmful effects of smoking on population health, the Government of Argentina approved a smoke-free law in 2011. The policy intends to reduce tobacco consumption, featuring a total ban on smoking in public settings, prohibition of advertising and promotional activities regarding tobacco use, and enforcing manufacturers to include messages warning of the harmful effects of cigarette smoking on health [12].

With the adoption of smoke-free policies in most developed countries, the general prevalence of smoking has declined, but rates remain particularly high among lower socio-economic groups, including those with lower educational levels, income, and employment status [13–17]. The higher smoking prevalence in the lowest socio-economic groups can partly explain the inequalities in health outcomes in most developed countries [14,18–20], where socio-economic inequalities in smoking contribute to inequalities in mortality [18,19]. Differences in smoking prevalence within population subgroups can translate into disparities in the tobacco-related burden of disease. Smoking is the single most important driver of health inequalities and is far more common among unskilled and low-income workers than among professional high earners [21]. Unprivileged groups bare a greater likelihood of smoking and suffering from smoking-related diseases and premature death. Tobacco use compounds existing social inequalities and poverty [22].

In many Latin American countries, socio-economic differences in morbidity and mortality are more pronounced than in developed nations, and tobacco may have a crucial role [23]. However, evaluation evidence on the equity impacts of tobacco control measures is still limited in comparison to the evidence base for other areas of tobacco control. Only a few studies have addressed socio-economic inequalities in smoking and its relation with health disparities in Argentina.

The present study aims to measure the extent and changes of socio-economic inequalities in smoking in the general population of Argentina from 2005 to 2013, and to assess socio-economic variables associated with involuntary exposure to tobacco smoke in 2005, 2009 and 2013. Understanding the pattern of socio-economic inequalities in tobacco consumption supports the design of targeted public health policies for tobacco control.

## Materials and methods

### Data and data sources

Data used for the present analysis were derived from the National Risk Factor Survey (NRFS) conducted in 2005, 2009 and 2013 by the Argentine National Ministry of Health (NMoH) and the National Statistics and Census Institute (INDEC). Data are available on an open access basis. A detailed description of the survey methodology and sampling method hasbeen published elsewhere [24,25]. Briefly, the NRFS is a nationally representative cross-sectional study that included persons aged 18 years and older, from the general population, using a multi-stage random sample of households in cities with more than 5000 inhabitants throughout the country. People residing in institutions, such as hospitals, retirement homes, nursing homes, or long-term care facilities, were not eligible for interviewing.

The response rate was 86.7% in 2005, 79.8% in 2009, and 70.7% in 2013, and the final number of participants was 41,392 in 2005; 34,372 in 2009; and 32,365 in 2013. In addition to a standard household questionnaire, an individual questionnaire was used to collect self-reported data on tobacco use and involuntary exposure to tobacco smoke. For the NRFS it was decided to carry out an adjustment to the initial weights through the technique of "calibration by fixed marginals" following the methodology developed by Deville and Särndal. More detail can be found in the methodological documents of each of the surveys [26–28].

### Smoking prevalence

The prevalence of smoking was defined as the proportion of people, aged 18 years and older, who responded that they currently smoke and have smoked more than 100 cigarettes in their lifetime [29].

### Involuntary exposure to tobacco smoke

The inhalation of tobacco smoke by nonsmokers has been variably referred to as “passive smoking”, “secondhand smoking” or “involuntary smoking”. In this study, we use the phrase“involuntary exposure to tobacco smoke” [30]. This variable was constructed from the population that does not currently smoke, that is, in the group of never-smokers and former smokers. According to this indicator, involuntary exposure to tobacco smoke was present when non-smokers reported that someone smoked in a closed place such home, workplace, educational institutions and bars or restaurants, hospitals or health care centers during the last 30 days.

### Socio-demographic variables

Socio-demographic variables were age, sex, and cohabitation. Age was defined as a categorical variable (18–24, 25–34, 35–49, 50–64, 65 and older). Two categories of cohabitation were used: living alone (single, divorced, separated or widowed) or living with someone (living as a couple or married).

### Socio-economic variables

As there is no single best indicator of SES, we selected three individual-level indicators to encompass the effect of socio-economic inequalities in smoking prevalence: educational level, household income per consumer unit, and employment status. Educational level is a frequently used indicator in epidemiological studies. Usually, it is established in early adulthood and remains stable [31]. It is relatively easy to measure in self-administered questionnaires and relevant to people regardless of age or working circumstances [32]. Although incomeis considered a sensitive issue and people may be reluctant to provide such information [33], it constitutes the SES indicator that most directly measures material resources. Finally, a link between employment and income has been established in many studies. However, there is also strong evidence that even after controlling for income, individuals who are unemployed have worse health outcomes than those who are employed [34].

Education level was defined as the highest level of education attained, based on the International Standard Classification of Education adopted by the UNESCO [35], and categorized into incomplete primary school or less, complete primary school and incomplete high school, complete high school or more. The total household income per consumer unit was calculated according to the criteria of the Organization for Economic Cooperation and Development (OECD) [36] using the equivalence scale equal to the square root of the number of household members. Subsequently, for the analysis, households were grouped in quintiles according to income per consumer unit (q1 the lowest, q5 the highest). Total household income was measuren in Argentine pesos and included income from work, pensions, rents, unemployment, insurance, scholarships, and food allowances. Employment status was defined as employed, unemployed (participants who do not have a job and have actively looked for work in the past four weeks) or inactive (participants who do not have a job and arenot looking for one).

### Statistical analysis and inequality measures

To achieve the first proposed goal, related to smoking in the population, a descriptive analysis was conducted to measure the smoking prevalence between 2005 and 2013 for each sex in relation to the following socio-economic factors: educational level, household income per consumer unit, and employment status.

Adittionally, two measures of inequality, the age-adjusted smoking prevalence ratio (PR) and the disparity index (DI), were used to calculate the magnitude and temporal trends of socio-economic inequalities in smoking for each sex. The PR for each SES variable was calculated using the highest SES group as the reference. Education level PR was calculated as the smoking prevalence for people with an incomplete primary education divided by the smoking prevalence for people with a complete high school education or more. Household income per consumer unit PR was defined as the smoking prevalence for people with the lowest quintile divided by the smoking prevalence for people with the highest quintile. Finally, the PR for employment status was defined as the smoking prevalence for unemployed people divided by the smoking prevalence for employed people. All PRs were adjusted by age using generalized linear models of family Poisson and a link function log [37,38]. The DI measures “the mean deviation of the group rates from some reference point (usually the best group rate) as a proportion of that reference point” [39,40]. The DI expresses the summed differences as a proportion of the reference rate. The total prevalence of smoking was used as the reference rate for each social group [39]. Thus, the DI was expressed as a percentage of the total smoking prevalence for the year analyzed. Given the multidimensional nature of health inequalities, it is not possible to summarize this concept in a single variable. However, using related and complementary measures of disparities, it is possible to capture differences in health outcomes that can be attributed to social determinants. The choice of these particular measures is because their main advantages. PR, a range measure which typically compares the two extreme categories, is easy to interpret while DI, a more complex relative measure, is sensitive to health differences between groups [41]. We need different indicators to capture different perspectives of the data, and the selected measures are complementary on this point.

To achieve the second proposed goal, related to involuntary exposure to tobacco smoke, generalized linear models (GLM), adjusted for age and sex, were used to examine the prevalence ratio (PR) for involuntary exposure to tobacco smoke in each year according to socio-economic variables [42]. Three models were built, using different independent socio-economic variables: (I) educational level; (II) quintiles by household income per consumer unit; and (III) employment status. Correlations and multicollinearity among the three socio-economic factors were analyzed; the correlation coefficients were <0.45, and multicollinearity was almost absent.

All statistical analyses were performed using Stata version 12 (Stata Corporation, College Station, Texas, USA). Given the complex survey design, estimates were weighted to more accurately reproduce national demographic characteristics. All results were presented with 95% confidence intervals (95% CI) and a p value <0.05 considered as significant.

### Ethics statements

The Ethics Committee of the Pan American Health Organization approved the NRFS. The database complies with National Law 17,622 of protection of statistical secret ensuring that the information presented is anonymous. The present study constitutes an analysis of aggregated secondary data that were obtained from the public domain on an open access basis, and does not allow the identification of the surveyed participants.

## Results

### Smoking prevalence trend between 2005 and 2013

Table 1 describes the smoking prevalences for Argentina in 2005, 2009 and 2013. The overall smoking prevalence in Argentina decreased from 29.7% in 2005 to 25.1% in 2013 (p <0.001). In men, the prevalence of smoking decreased from 35.1% in 2005 to 29.9% in 2013 (p <0.001). However, this trend was significant only for age groups younger than 50 years, and more marked in men aged 35–49 years (p <0.001). In women, smoking prevalence decreased from 24.9% in 2009 to 20.9% in 2013. The main reduction in smoking rates was seen for women of 18–24 years (p <0.001) followed by women of 35–49 years (p <0.001). In women aged 65 years and older, a noticeable increase of the smoking prevalence was observed (p <0.001).

**Table 1:** Smoking weighted prevalence by selected characteristics—National Risk Factors Survey, Argentina, 2005, 2009 and 2013.

	2005						2009						2013					
Variable	Overall (n = 41392)		Men (n = 17827)		Women (n = 23565)		Overall (n = 34732)		Men (n = 15028)		Women (n = 19704)		Overall (n = 32365)		Men (n = 14317)		Women (n = 18048)	
	%	95% CI	%	95% CI	%	95% CI	%	95% CI	%	95% CI	%	95% CI	%	95% CI	%	95% CI	%	95% CI
**All**	29.7	(28.7–30.8)	35.1	(33.5–36.7)	24.9	(23.6–26.2)	27.1	(26.4–27.9)	32.4	(31.2–33.7)	22.4	(21.5–23.4)	25.1	(24.2–26.1)	29.9	(28.4–31.4)	20.9	(19.7–22.1)
**Age (years)**																		
18–24	36.1	(33.4–38.9)	39.9	(36.0–44.0)	31.8	(28.3–35.6)	28.8	(26.7–30.9)	33.5	(30.4–36.9)	24.0	(21.4–26.7)	26.7	(24.0–29.5)	32.4	(28.4–36.7)	20.9	(17.6–24.6)
25–34	34.6	(32.4–36.9)	43.0	(39.5–46.6)	27.3	(24.7–30.2)	33.3	(31.6–35.1)	39.4	(36.7–42.2)	27.3	(25.2–29.5)	30.8	(28.7–33.0)	36.2	(33.0–39.5)	25.5	(22.8–28.3)
35–49	35.8	(33.8–38.0)	39.8	(36.8–42.8)	32.4	(29.5–35.3)	30.3	(28.9–31.9)	35.4	(33.1–37.8)	26.1	(24.3–28.1)	26.7	(25.0–28.5)	30.1	(28.3–33.8)	22.7	(20.4–25.1)
50–64	26.8	(24.7–29.0)	31.3	(28.0–34.7)	22.4	(20.0–25.1)	27.9	(26.3–29.7)	32.4	(29.7–35.1)	24.2	(22.2–26.4)	26.2	(24.2–28.3)	30.1	(27.0–33.5)	22.7	(20.3–25.3)
≥ 65	8.9	(7.7–10.3)	12.9	(10.6–15.7)	6.0	(4.9–7.4)	10.2	(9.1–11.4)	14.6	(12.5–17.0)	6.9	(5.8–8.2)	11.2	(9.7–12.9)	12.4	(10.0–15.2)	10.4	(8.5–12.7)
**Cohabitation**																		
Living alone	31.0	(29.3–32.6)	36.2	(33.5–39.0)	27.2	(25.3–29.3)	27.6	(26.4–28.9)	34.4	(32.4–36.6)	22.3	(21.0–23.7)	26.2	(24.8–27.7)	31.7	(29.3–34.2)	21.9	(20.3–23.7)
Living with someone	28.9	(27.7–30.2)	34.4	(32.5–36.4)	23.1	(21.5–24.8)	63.2	(25.8–27.7)	31.2	(29.7–32.7)	22.5	(21.3–23.8)	24.0	(23.2–25.6)	28.7	(26.9–30.6)	20.0	(18.5–21.7)
**Education level**																		
Incomplete primary	23.6	(21.1–26.6)	31.0	(27.0–35.2)	18.5	(15.3–22.1)	23.0	(21.0–25.1)	32.6	(29.1–36.3)	15.8	(13.7–18.2)	21.6	(18.9–24.6)	26.5	(22.3–31.1)	17.4	(14.0–21.5)
Incomplete High School	31.5	(29.8–33.1)	38.4	(36.0–40.9)	24.5	(22.4–26.7)	29.9	(28.6–31.1)	36.4	(34.4–38.3)	23.5	(22.0–25.1)	29.7	(28.1–31.4)	35.4	(32.9–37.9)	23.9	(21.7–26.1)
Complete High School or more	29.9	(28.3–31.3)	32.6	(30.4–35.0)	27.4	(25.6–29.2)	25.8	(24.7–26.9)	29.0	(27.2–30.7)	23.1	(21.8–24.5)	22.5	(21.3–23.7)	26.0	(24.1–28.0)	19.5	(18.1–21.0)
**Household income per consumer**																		
I(Lowest)	33.1	(30.9–35.4)	43.7	(40.1–47.4)	24.8	(22.3–27.5)	30.4	(28.8–32.1)	38.7	(36.1–41.4)	23.8	(21.9–25.7)	28.2	(26.2–30.4)	34.8	(31.4–38.4)	23.0	(20.6–25.5)
II	26.6	(24.3–28.9)	32.1	(28.8–35.7)	22.1	(19.2–25.4)	25.9	(24.2–27.6)	33.0	(30.3–35.8)	19.9	(18.0–21.9)	25.2	(23.1–27.4)	28.8	(25.7–32.2)	22.2	(19.5–25.2)
III	31.4	(29.1–33.9)	36.1	(32.7–39.7)	26.9	(23.9–30.1)	24.3	(22.7–26.0)	30.1	(28.1–33.7)	18.7	(16.9–20.7)	25.2	(23.0–27.4)	31.2	(27.8–34.8)	19.2	(16.7–21.9)
IV	28.6	(26.4–30.9)	32.8	(29.3–36.6)	24.4	(21.9–27.1)	27.0	(25.2–28.8)	29.5	(26.8–32.3)	24.7	(22.4–27.1)	23.8	(21.7–26.0)	28.0	(24.9–31.4)	20.2	(17.6–23.0)
V(Highest)	27.7	(25.7–29.9)	28.4	(25.5–31.6)	27.0	(24.2–30.1)	27.3	(25.5–29.2)	29.3	(26.6–32.2)	25.3	(23.0–27.8)	23.0	(21.1–25.0)	26.5	(23.7–29.6)	19.3	(16.9–21.9)
**Employment status**																		
Unemployed	34.1	(29.6–39.0)	33.7	(27.2–40.8)	34.4	(28.4–41.1)	34.3	(30.4–38.4)	38.5	(32.2–45.2)	30.9	(26.1–36.2)	34.0	(29.0–39.3)	35.5	(28.3–43.5)	32.6	(26.1–39.8)
Inactive	17.6	(16.3–19.1)	20.5	(17.8–23.6)	16.6	(15.1–18.3)	17.6	(16.5–18.7)	20.3	(18.1–22.8)	16.5	(15.3–17.7)	16.4	(15.0–17.8)	18.0	(15.4–20.9)	15.8	(14.2–17.5)
Employed	35.4	(34.1–36.8)	38.3	(36.5–40.2)	31.3	(29.4–33.2)	31.4	(30.4–32.4)	35.1	(33.6–36.5)	26.6	(25.2–28.0)	29.1	(27.9–30.4)	32.5	(30.9–34.3)	24.5	(22.8–26.2)

Smoking prevalence was defined as the proportion of people, aged 18 years and older, who responded that they currently smoke and have smoked more than 100 cigarettes in their lifetime.

### Socio-economic variations of smoking prevalence between 2005 and 2013

Regarding educational level, a sharp decrease in smoking prevalence was only observed among the most educated population (-24.7%; p <0.001; Table 1), trend that stands for men (-20.2%, p <0.001) and women (-28.8%, p <0.001). The change in smoking prevalence based on household income per consumer unit showed a reduction in smoking rates for women in the highest three quintiles (Table 1), while for men a significant reduction was seen only in the first quintile (-20.4%; p <0.001). Regarding employment status, the smoking prevalence had a statistically significant reduction only for the employed population, in both men and women (-15.1%; p <0.001 and -21.7%; p <0.001 respectively; Table 1).

### Inequalities in smoking prevalence

Table 2 shows the change in smoking prevalence disparities from 2005 to 2013 for populations defined by educational level, quintils of household income per consumer unit and employment status. In men, a consistently elevated PR across the three surveys can be seen for educational level and household income consumer unit, meaning that population with lower educational level or household income per consumer unit exhibit higher age-adjusted smoking prevalence. In women, only employment status in the 2013 survey showed a signficantly elevated PR (1.32; 95% CI: 1.06, 1.66). The DI is moderately high for smoking prevalence in both sexes (range: 14.47%-33.06%). Disparity increased slightly but was highly variable within a narrow range of values across the studied years.

**Table 2:** Socioeconomic inequalities in smoking prevalence—National Risk Factors Survey, Argentina, 2005, 2009 and 2013.

	Men			Women		
Inequalities metrics	2005 (n = 17827)	2009 (n = 15028)	2013 (n = 14317)	2005 (n = 23565)	2009 (n = 19704)	2013 (n = 18048)
**Prevalence Ratio**^a^ **(95% CI)**						
Education level (incomplete primary vs. complete secondary and more)	**1.24 (1.07–1.43)**	**1.39 (1.23–1.57)**	**1.23 (1.03–1.48)**	0.95 (0.79–1.15)	0.92 (0.79–1.08)	1.15 (0.91–1.45)
Income (lowest quintile vs. highest quintile)	**1.51 (1.32–1.73)**	**1.32 (1.17–1.48)**	**1.27 (1.09–1.47)**	0.87 (0.75–1.01)	0.92 (0.82–1.04)	1.17 (0.98–1.38)
Employment status (unemployed vs. employed)	0.87 (0.70–1.07)	1.11 (0.93–1.32)	1.06 (0.85–1.33)	1.05 (0.86–1.29)	1.16 (0.97–1.38)	**1.32 (1.06–1.66)**
**Disparity Index**						
Education level^b^	14.47	20.50	16.73	20.98	23.24	19.25
Income^c^	18.31	18.52	17.84	15.69	17.04	17.21
Employment status^d^	24.46	32.22	33.06	21.77	18.32	23.10

Abbreviations: CI, confidence interval.

Smoking prevalence was defined as the proportion of people, aged 18 years and older, who responded that they currently smoke and have smoked more than 100 cigarettes in their lifetime.

^a^ Adjusted by age.

^b^ Categories: incomplete Primary; incomplete High School; complete High School or more.

^c^ Quintiles of household income per consumer.

^d^ Categories: Unemployed; inactive (respondent does not have a job and is not looking for one); employed.

### Involuntary exposure to tobacco smoke

**Fig 1:**
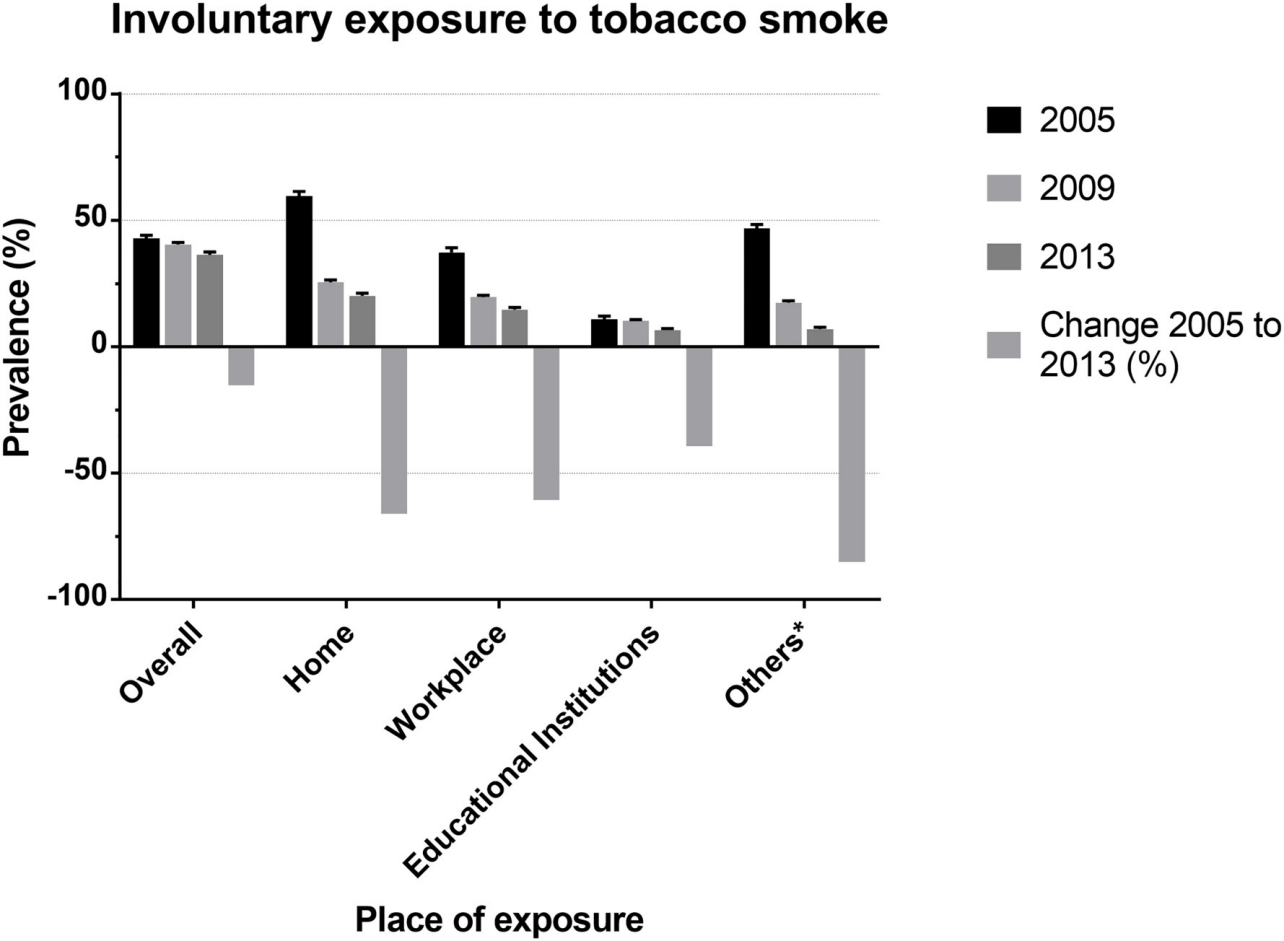
Prevalence of involuntary exposure to tobacco smoke in adults from argentina by place of exposure.

Overall involuntary exposure to tobacco smoke (Fig 1) has decreased by 15.2%, from 42.8% in 2005 to 36.3% in 2013 (p <0.001), with the greater reduction attributable to bars or restaurants and hospitals or healthcare centers,where the prevalence decreased by 85.0%, from 46.8% in 2005 to 7% in 2013 (p <0.001). S1 Table describes specific frequencies for each year.

In all GLM analyses performed for the three surveys, age and cohabitation were co-variables significantly associated with secondhand smoking (Table 3). A gradient can be seen towards a reduction in involuntary exposure to tobacco smoke for older age groups compared to the youngest age group. People living with someone were less likely to be involuntarily exposed to tobacco smoke.

**Table 3:** Prevalence ratio (PR) from generalized linear models (GLM), with 95% confidence intervals, on the PR of involuntary exposure to tobacco smoke for socioeconomic and individual factors amongst adults. National Risk Factors Survey, Argentina, 2005, 2009 and 2013.

	2005 (n = 41392)			2009 (n = 34732)			2013 (n = 32365)		
Variable	Model 1: Education Level	Model 2: Income	Model 3: Employment	Model 1: Education Level	Model 2: Income	Model 3: Employment	Model 1: Education Level	Model 2: Income	Model 3: Employment
	PR (95% CI)	PR (95% CI)	PR (95% CI)	PR (95% CI)	PR (95% CI)	PR (95% CI)	PR (95% CI)	PR (95% CI)	PR (95% CI)
**Age (years)**									
18–24	1	1	1	1	1	1	1	1	1
25–34	**0.82 (0.75–0.90)**	**0.83 (0.76–0.90)**	**0.80 (0.73–0.87)**	**0.87 (0.81–0.93)**	**0.86 (0.81–0.93)**	**0.84 (0.78–0.90)**	**0.85 (0.78–0.94)**	**0.85 (0.78–0.93)**	**0.82 (0.75–0.90)**
35–49	**0.68 (0.62–0.74)**	**0.68 (0.62–0.75)**	**0.66 (0.60–0.72)**	**0.63 (0.58–0.67)**	**0.63 (0.59–0.68)**	**0.61 (0.57–0.66)**	**0.65 (0.59–0.72)**	**0.66 (0.60–0.73)**	**0.64 (0.58–0.71)**
50–64	**0.63 (0.57–0.70)**	**0.65 (0.59–0.71)**	**0.63 (0.57–0.70)**	**0.57 (0.53–0.62)**	**0.60 (0.55–0.65)**	**0.58 (0.54–0.63)**	**0.59 (0.53–0.66)**	**0.63 (0.56–0.70)**	**0.61 (0.55–0.68)**
> = 65	**0.40 (0.36–0.45)**	**0.41 (0.37–0.47)**	**0.44 (0.39–0.49)**	**0.40 (0.37–0.44)**	**0.43 (0.39–0.47)**	**0.45 (0.41–0.49)**	**0.40 (0.35–0.45)**	**0.44 (0.39–0.49)**	**0.44 (0.39–0.50)**
**Sex**									
Men	1	1	1	1	1	1	1	1	1
Women	**0.90 (0.85–0.96)**	**0.90 (0.85–0.96)**	0.94 (0.89–1.00)	0.95 (0.91–1.00)	**0.95 (0.90–0.99)**	0.98 (0.93–1.03)	0.94 (0.88–1.00)	**0.93 (0.87–0.99)**	0.95 (0.89–1.02)
**Cohabitation**									
Living alone	1	1	1	1	1	1	1	1	1
Living with someone	**0.88 (0.82–0.93)**	**0.88 (0.82–0.94)**	**0.88 (0.83–0.94)**	**0.91 (0.86–0.95)**	**0.92 (0.87–0.96)**	**0.92 (0.87–0.97)**	**0.84 (0.78–0.90)**	**0.85 (0.79–0.91)**	**0.85 (0.79–0.91)**
**Educational level**									
Incomplete primary school or less	1			1			1		
Incomplete high school	0.95 (0.86–1.06)			**0.92 (0.85–0.99)**			**0.88 (0.79–0.98)**		
Complete high school or more	0.92 (0.83–1.02)			**0.80 (0.74–0.87)**			**0.73 (0.65–0.81)**		
Quintiles by household income per consumer unit*									
I (Lowest)		1			1			1	
II		1.01 (0.92–1.10)			1.02 (0.96–1.09)			0.98 (0.89–1.08)	
III		0.94 (0.85–1.04)			0.94 (0.88–1.01)			0.98 (0.89–1.07)	
IV		0.97 (0.88–1.07)			0.96 (0.89–1.03)			**0.89 (0.80–0.98)**	
V (Highest)		0.99 (0.90–1.08)			**0.91 (0.84–0.98)**			**0.84 (0.76–0.93)**	
**Employment status**									
Unemployed			1			1			1
Inactive			0.87 (0.76–1.00)			0.90 (0.80–1.00)			0.90 (0.78–1.04)
Employed			1.02 (0.90–1.16)			1.01 (0.91–1.12)			0.98 (0.85–1.13)

Abbreviations: PR, prevalence ratio; CI, confidence interval.

Model 1: Educational level was considered as the independent socio-economic variable.

Model 2: Quintil by household income per consumer unit was considered as the independent socio-economic variable.

Model 3: Employment status. was considered as the independent socio-economic variable.*Using the Square Root Scale, which divides household income by the square root of household size.

Bolded values stand for statistically significant findings at <0.05 level.

While educational level (model 1) was not associated with secondhand smoking in 2005, a significantly lower prevalencce for the higher educational level is observed in 2009, which is accentuated in 2013.The population with the higher educational level had a 0.73-fold lower prevalence ratio (PR, 0.73; 95% CI: 0.65, 0.81) of being involuntarily exposed to tobacco in 2013 than did those people in the lower educational level. A similar association was observed in 2009 (PR, 0.80; 95% CI: 0.74, 0.87).

A comparable pattern was observed for quintiles of household income per consumer unit (model 2). While no association was found in 2005, a statistically significant lower prevalence of involuntary exposure to tobacco smoke was present among the higher income quintiles in 2009 and 2013.

In model 3, employment status was not associated with involuntary exposure to tobacco smoke in any of the years of the survey.

## Discussion

The main finding of our study is that, although the prevalence of tobacco smoking has declined in Argentina over the last years, from 2005 through 2013, socio-economic inequalities in smoking habits have persisted. Moreover, in recent surveys, key socioeconomic factors such as educational level and household income exhibited increased socioeconomic inequalities in secondhand smoking.

### Changes in smoking prevalence and socio-economic inequalities in smoking

According to our study, the proportion of smokers in Argentina has decreased by 15.5% over a relatively short period of time, from 29.7% in 2005 to 25.1% in 2013. This trend is similar to that of other countries in the region [43–46]. According to the Global Burden of Disease report, the proportion of smokers between 2005 and 2015 dropped in all South American countries, except in Paraguay, where the prevalence of smokers increased by almost one point only among women [46].

These changes occurred in the context of the adoption and implementation of a set of best practices disclosed by the WHO as tobacco protective, some of which have been implemented in Argentina [47].Even though Argentina has not ratified the WHO Framework Convention on Tobacco Control (FCTC), which the country signed in 2003, the National Ministry of Health (NMoH) and civil society groups have worked effectively over the last years in the fight against tobacco: they have built coalitions, disseminated information, and helped deliver health services to help individuals stop smoking [48]. In particular, they played a key role in the approval of the National Tobacco Control Law in 2011 [49].

In other countries in the region (i.e.: Brazil, Uruguay, and Panama), tobacco control policies, such as the complete ban on tobacco advertising, were initiated earlier. The decline in the proportion of smokers in some of these countries, for example, Brazil, was much more marked than in Argentina [46].

Studies conducted in many countries have reported that socio-economic differences in the prevalence of smoking among men and women based on variables persisted over time [20,23,50,51]. Notwithstanding the reduction in smoking prevalence in Argentina, our results show that socio-economic inequalities persisted over the studied period. As we have seen, the decrease in the prevalence of smoking was only seen among those adults who have a higher educational attainment. Moreover, the decrease in smoking prevalence was at the expense of the groups with employment and with a higher household income.

Our results indicate that, while overall reductions in smoking prevalence are occurring, they are unevenly distributed in SES levels, thus increasing disparities in smoking prevalence.In this scenario, it is feasible to expect a parallel unequal burden of smoking-related adverse health outcomes.

A similar pattern has been described in other countries (United States, Netherlands, England., Australia) [52–56]. Many studies have identified that smokers from lower socio-economic groups are less likely to be successful in stopping smoking than more affluent smokers, even after accessing cessation programs [57,58]. Link and Phelan proposed a conceptual discussion of such pattern [59]. When major disease processes, in this case tobacco habits, move from processes that humans cannot control to processes that individuals understand and do control, at least to some extent, a social shaping of health disparities occurs. When people learn about tobacco and its very harmful health consequences they are immediately engaged and determine whether and to what extent the health enhancing information travels through the population. Groups with greater resources of knowledge, money, power prestige and beneficial social connections generally benefit more, producing disparities by SES.

The reason why people from lower socio-economic groups smoke more remains a complex question that requires further research. Indeed, the fact that tobacco is consumed more by people for whom it is, in relative terms, more expensive, is paradoxical [60]. We can conceivably hypothesize that the poor and less educated are less aware of the health hazards of smoking and thus more likely to adopt this harmful practice. Others argue that smoking may be a self-medication used to regulate mood, manage stress, and to cope with the strains of material deprivation [61,62]. At last, economic hypotheses suggest that, given the same perceived benefits from smoking, a person whose income is low would have less to lose from future health problems than a person with a higher income [60].

It is important to put in context our results in relation to social and demographic changes occurring in the population over time [42]. Argentina entered the 21st century mired in a deep socioeconomic and political crisis [63]. In 2001, Argentina experienced the worst economic depression in the country’s history with over 50% of its population living in poverty. Regarding demographic changes, the total population reached 41,223,000 in 2010, compared to 39,145,000 in 2005 [64]. However, for the same period, the proportion of people between 15 and 65 years of age remained close to 63%, and 10% for those 65 and older [64].

Finally, our study suggest that smoking inequality (PR) in men is associated with two socio-economic parameters (education and income), while no such association is observed in women. Even though Table 1 shows consistent sex differences in the prevalence of smoking by socio-economic and socio-demographic variables, such differences are not observed in women after the age-adjustment. It is also important to interpret differences in the results obtained from the analyses of the two selected inequality metrics (PR and DI). For example, while PR for employment status are almost all insignificant, the DI attributed to employment are the highest for almost all years for both men and women. This situation is problaby because each measure looks upon different aspects of the problem. PR considers the difference between the extremes, while DI contemplates the mean dispersion of the data.The DI is a complex, unweighted measure of inequality that shows the proportional difference between each subgroup and the national level, on average. DI is calculated for non-ordered dimensions. It takes only positive values with larger values indicating higher levels of inequality [65]. So, it is important to enrich our understanding of the processes behind inequality and changes in inequality, and to bring this to the forefront of policy debate. This includes developing a more multidimensional perspective on inequality, but also other aspects such as considering inequality at different levels of aggregation and different time horizons [66]. WHO enables the assessment of inequalities using multiple summary measures [67]. Public health policy has recognised the growing importance of socioeconomic determinants of health, such as income, education, employment, housing and the environment, as well as their effect on lifestyle [68]. To understand smoking inequality itself and to develop strategies to reduce smoking disparities, knowledge of the underlying principles or mechanisms of the inequality over a long time-course may be important [69]. Re-evaluation of the impact of the interventions on smoking inequality using a long time-course perspective may lead to a favorable next step in equity effectiveness. Tackling socioeconomic inequality in smoking may be a key public health target for the reduction of inequality in health [69].

### Socio-economic inequalities in involuntary exposure to tobacco smoke

Involuntary exposure to tobacco smoke has decreased, but our results indicate that in 2013 both educational level and household income were associated with secondhand smoking. A range of studies have found evidence of inequities in involuntary exposure to tobacco smoke, with minorities generally experiencing higher risks [70,71]. A recent systematic review in 15 low and middle-income countries (LMICs) concluded that tobacco exposure at home is higher among the socio-economically disadvantaged in the majority of the LMICs studied. Similarly, tobacco exposure at workplaces is higher among the less educated [71]. In Argentina, although the smoking ban law is national, with the aim of being applied throughout the territory, and seeks to reduce inequities, disparities still persist.

### Limitations and strengths

Our study has some limitations. First, disparities are examined from a broad perspective, since there is no unique indicator that can comprehensively address the issue. However, PR and DI are adecuate to estimate the magnitude and temporal trends of socio-economic inequalities and were used and recommended in the literature [20,39,40]. Second, the surveys did not include rural population or people residing in institutions. Further studies which approach these specific populations would help build a more comprehensive picture of health disparities in any given country. Third, the surveys are limited to adults aged 18 years and older. Further analytic approaches could include a younger population to explore how the social gradients in smoking prevalence and involuntary exposure to tobacco smoke could be influenced by socioeconomic factors [72]. Another important point is that data used from surveys are annual and representative of the Argentine population; that is to say that even with the limitations of a cross-sectional survey, we obtained the characteristics of a population at a given time and under the circumstances that the population lived at that time. In this sense, we consider that our results reflect what happened in Argentina each year and allow an approximation of the behavior of inequalities year after year, without being a longitudinal analysis.

The main strength of our study is that we used nationally representative surveys, with repeated measurements over time and a high response rate. Additionally, it is important to highlight that the results of our study may have important implications in terms of policy making and development of intervention strategies to promote smoking cessation in the general population. The findings of our analysis indicate that tobacco control policy and public health interventions need to consider widespread socio-economic inequities in tobacco consumption.

## Conclusions

Although overall smoking rate has decreased in Argentina over the last years, disparities related to tobacco smoking by SES still persist. Vulnerable groups still have a higher smoking prevalence. Comprehensive tobacco control programs are essential in developing strategies to reduce health disparities in tobacco-related diseases. Health inequalities can be reduced through measures that have a greater effect on smokers in higher prevalence groups. This means both prioritizing population-level interventions to which disadvantaged smokers are more sensitive, and targeting interventions to these population. Public health policies for tobacco control should prioritize strategies aimed at narrowing the health disparity gap.

## Supporting information

S1 Table Involuntary exposure to tobacco smoke between 2005 and 2013. (DOCX)
