# Supplementary material for: Socio-economic inequalities in smoking prevalence and involuntary exposure to tobacco smoke in Argentina: Analysis of three cross-sectional nationally representative surveys in 2005, 2009 and 2013
Source: PLoS One. 2019 Jun 7;14(6):e0217845. doi: 10.1371/journal.pone.0217845 (PMC6555547; doi:10.1371/journal.pone.0217845)
Supplement: S1 Table — (DOCX) [file pone.0217845.s001.docx]

S1 Table. Involuntary exposure to tobacco smoke between 2005 and 2013.

| **Involuntary exposure to tobacco smoke** | **2005** |  | **2009** |  | **2013** |  | **Change 2005 to 2013** |  |
| --- | --- | --- | --- | --- | --- | --- | --- | --- |
|  | **Prevalence** | **95% CI** | **Prevalence** | **95% CI** | **Prevalence** | **95% CI** | **%** | **p value** |
| Overall | 42.8 | (41.5 - 44.1) | 40.4 | (39.4 - 41.3) | 36.3 | (35.1 - 37.5) | -15.2 | <0.001 |
| Home | 59.5 | (57.6 - 61.4) | 25.5 | (24.6 - 26.4) | 20.2 | (19.2 - 21.2) | -66.1 | <0.001 |
| Work place | 37.3 | (35.4 - 39.2) | 19.6 | (18.8 - 20.4) | 14.7 | (13.9 - 15.6) | -60.6 | <0.001 |
| Educationalinstitution | 10.9 | (9.6 - 12.1) | 10.2 | (9.6 - 10.8) | 6.6 | (6.0 - 7.2) | -39.4 | <0.001 |
| Others* | 46.8 | (44.8 - 48.8) | 17.4 | (16.6 - 18.2) | 7.0 | (6.4 - 7.7) | -85.0 | <0.001 |

Source: Argentina’s National Risk Factor Survey 2005, 2009 and 2013.

*Others: bar/restaurants, hospitals/health care centers
